# Supplementary material for: Enhanced dietary reconstruction of Korean prehistoric populations by combining δ13C and δ15N amino acids of bone collagen
Source: PLoS One. 2024 Mar 27;19(3):e0300068. doi: 10.1371/journal.pone.0300068 (PMC10971582; doi:10.1371/journal.pone.0300068)
Supplement: S1 File — (PDF) [file pone.0300068.s001.pdf]

Supplementary Information

Enhanced dietary reconstruction of Korean prehistoric populations by combining $\delta^{13}C$ and $\delta^{15}N$ amino acids of bone collagen

Kyungcheol Choy[1]*, Hee Young Yun[2], Benjamin T. Fuller[3], Marcello Mannino[3]

[1]*Department of Cultural Anthropology, Hanyang University ERICA, Ansan, South Korea*
[2]*Department of Marine Sciences and Convergence Engineering, Hanyang University ERICA, Ansan, South Korea*
[3]*Department of Archaeology and Heritage Studies, School of Culture and Society, Aarhus University, Højbjerg, Denmark*

The PDF files includes:

Fig. S1-1,2,3
Tables S1 to S6
R Code Documents 1 to 3

Other supplementary materials for this manuscript including the following:
1. Data file for PCA analysis (excel file)
2. Data file for bulk MixSIAR model (excel file)
3. Data file for AA MixSIAR model (excel file)
These data files are uploaded separately.

1. Food sources only

Option1: EAA N+ EAA

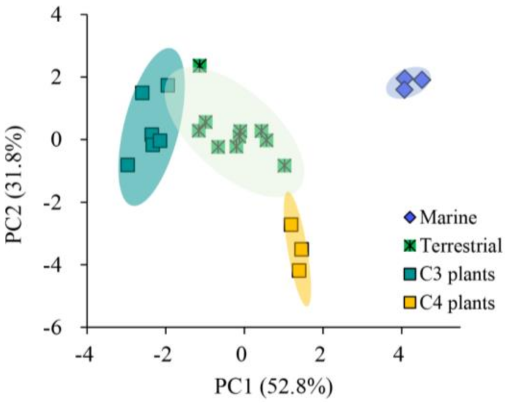

Option2: Three source AA + EAA

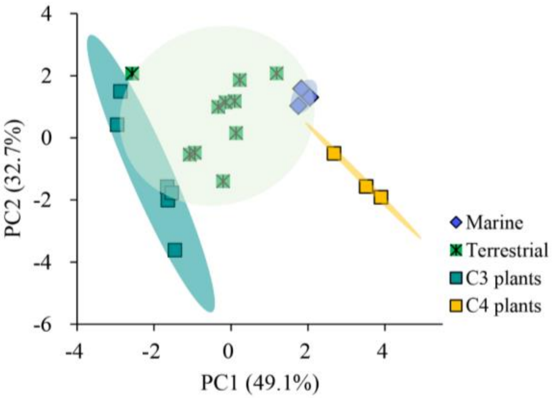

Option3: One source AA +EAA

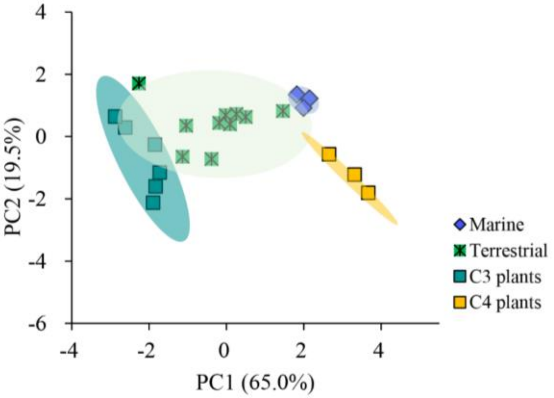

2. Imdang Humans

Option1: EAA N+ EAA

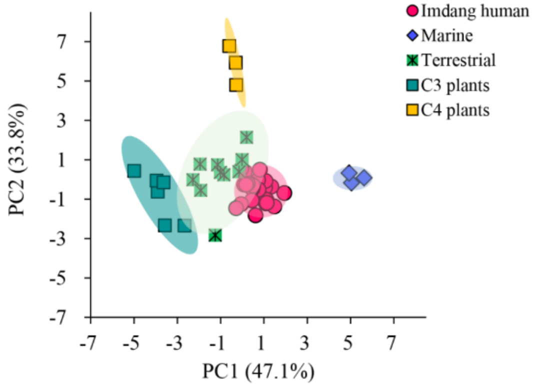

Option2: Three source AA + EAA

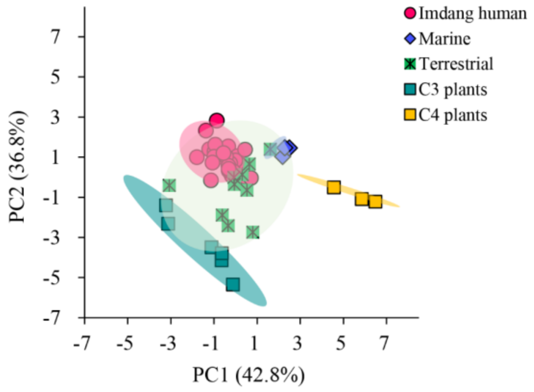

Option3: One source AA +EAA

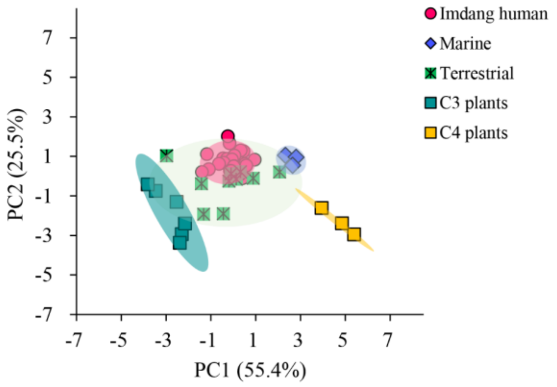

3.Mumun Humans

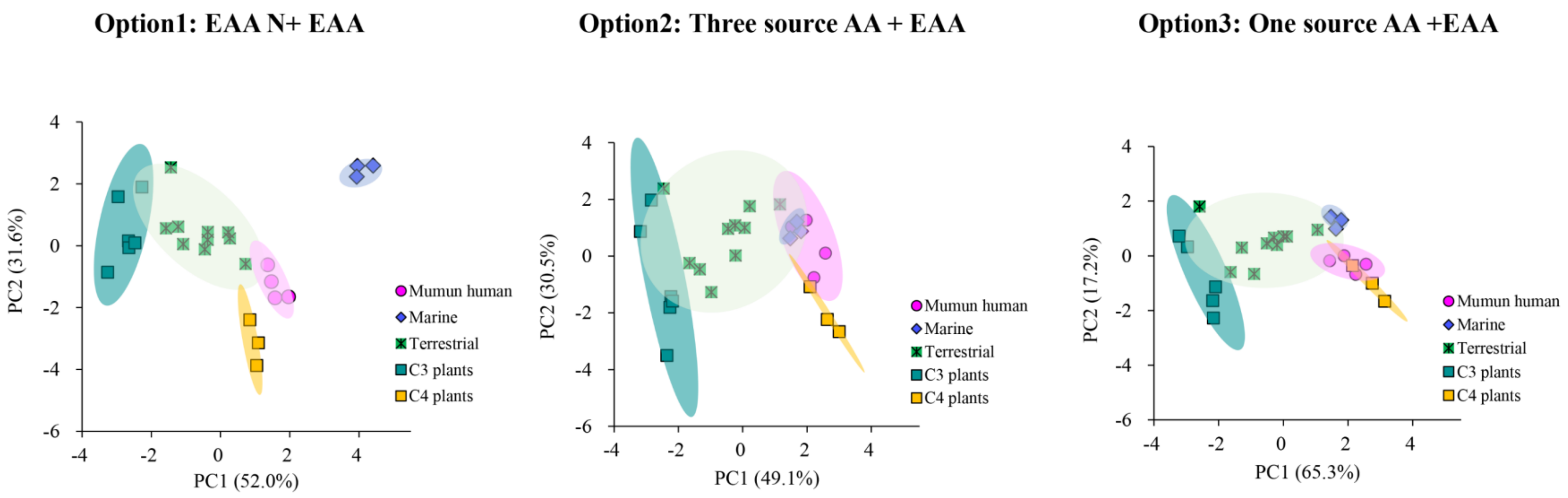

Supplementary Figure 1. Three score plots of principal component analyses (PCA) of δ^{13}C and δ^{15}N values of food sources and humans (Imdang and Mumun). To assess differences in food groups, we carried out principal component analyses (PCA) using three options: **Option 1**. Five EAA δ^{13}C (Phe, Leu, Val, Ile, Thr) values and four EAA δ^{15}N (Val, Leu, Ile, Phe) values; **Option 2**. Five EAA δ^{13}C (Phe, Leu, Val, Ile, Thr) values and three source δ^{15}N AAs (Ser, Gly, Phe) values; **Option 3**. Five δ^{13}C (Phe, Leu, Val, Ile, Thr) values and one source AA (Phe). Ellipses represent 95% standard deviation with arrows for EAAs being significant ($p<0.05$) correlation vectors. Of the three PCA options, Option 1 most effectively separates and cluster each food source: Marine, Terrestrial, C_3 and C_4 plants.

Supplementary Tables

Supplementary Table 1. Information (A) and stable carbon (B) and nitrogen (C) values of amino acids and bulk collagen of the human remains analyzed in this study.

A. Sample Information

Sample ID	Location	Species	Element	Burial	Type	Sex	Age	Chronology
ERI-HW1	Jecheon Hwangsok-ri	*Homo sapiens*	Femur	Stone Cist	Commoner	NA	NA	Middle Mumun (BC 566)
ERI-HW3	Jecheon Hwangsok-ri	*Homo sapiens*	Cranium	Stone Cist	Commoner	NA	NA	Middle Mumun (BC 566)
ERICA-KO8	Chuncheon Jungdo	*Homo sapiens*	Cranium	Stone Cist	Commoner	NA	NA	Middle Mumun (BC 566)
ERICA-JE9	Jeongseon MaeDun	*Homo sapiens*	Rib	NA	Commoner	NA	NA	Middle Mumun (BC 566)
SID-04	Joyeong EⅠ-2ho-1	*Homo sapiens*	Phalange	Double Wooden chamber	Retainer	NA	6-10	Proto-Three Kingdom (BC 80- 394 AD)
SID-05	Joyeung EⅠ-2ho-2	*Homo sapiens*	Cranium	Double Wooden chamber	Elite	NA	36-50	Proto-Three Kingdom (BC 80- 394 AD)
SID-07	Joyeung EⅡ-1ho-1	*Homo sapiens*	Femur	Stone Chamber	Elite	NA	Adult	Proto-Three Kingdom (BC 80- 394 AD)
SID-08	Joyeung EⅡ-1ho-2	*Homo sapiens*	Femur	Stone Chamber	Retainer	NA	Adult	Proto-Three Kingdom (BC 80- 394 AD)
SID-09	Joyeung EⅡ-2ho-1	*Homo sapiens*	Cranium	Double Wooden chamber	Retainer	NA	15-18	Proto-Three Kingdom (BC 80- 394 AD)
SID-10	Joyung EⅡ-2ho-2	*Homo sapiens*	Femur	Double Wooden chamber	Retainer	NA	Adult	Proto-Three Kingdom (BC 80- 394 AD)
SID-11	Joyung EⅡ-2ho-3	*Homo sapiens*	Rib	Double Wooden chamber	Retainer	NA	7.5-12.5	Proto-Three Kingdom (BC 80- 394 AD)
SID-12	Joyeung EⅡ-2ho-4	*Homo sapiens*	Femur	Double Wooden chamber	Elite	NA	21-35	Proto-Three Kingdom (BC 80- 394 AD)
SID-13	Joyeung EⅡ-2ho-5	*Homo sapiens*	Femur	Double Wooden chamber	Retainer	M	Adult	Proto-Three Kingdom (BC 80- 394 AD)
SID-15	Joyeung EⅡ-3ho-1	*Homo sapiens*	Femur	Double Wooden chamber	Elite	NA	21-35	Proto-Three Kingdom (BC 80- 394 AD)
SID-16	Joyeung EⅡ-3ho-2	*Homo sapiens*	Fibula	Double Wooden chamber	Retainer	NA	20	Proto-Three Kingdom (BC 80- 394 AD)
SID-17	Joyeung EⅡ-3ho-3	*Homo sapiens*	Cranium	Double Wooden chamber	Retainer	F	Adult	Proto-Three Kingdom (BC 80- 394 AD)
SID-19	Joyeung EⅡ-4ho-1	*Homo sapiens*	Femur	Double Wooden chamber	Elite	NA	NA	Proto-Three Kingdom (BC 80- 394 AD)
SID-20	Joyeung EⅡ-5ho-1	*Homo sapiens*	Fibula	Stone Chamber	Elite	M	Adult	Proto-Three Kingdom (BC 80- 394 AD)
SID-21	Joyeung EⅡ-6ho-1	*Homo sapiens*	Humerus	Double Wooden chamber	NA	F	21-35	Proto-Three Kingdom (BC 80- 394 AD)
SID-22	Joyeung EⅡ-7ho-1	*Homo sapiens*	Humerus	Double Wooden chamber	Elite	M	21-40	Proto-Three Kingdom (BC 80- 394 AD)
SID-25	Joyeung EⅡ-2ho-2	*Homo sapiens*	Rib	Double Wooden chamber	Retainer	M	21-35	Proto-Three Kingdom (BC 80- 394 AD)
SID-26	Joyeung EⅡ-2ho-3	*Homo sapiens*	Rib	Double Wooden chamber	Retainer	NA	6-12	Proto-Three Kingdom (BC 80- 394 AD)
SID-27	Joyeung EⅡ-2ho-4	*Homo sapiens*	Rib	Double Wooden chamber	Retainer	F	21-35	Proto-Three Kingdom (BC 80- 394 AD)
SID-28	Joyeung EⅡ-2ho-5	*Homo sapiens*	Rib	Double Wooden chamber	Retainer	M	21-35	Proto-Three Kingdom (BC 80- 394 AD)
SID-29	Joyeung EⅢ-3ho-1	*Homo sapiens*	Humerus	Double Wooden chamber	Elite	NA	Adult	Proto-Three Kingdom (BC 80- 394 AD)

SID-30	Joyeung EⅢ-3ho-2	*Homo sapiens*	Humerus	Double Wooden chamber	Retainer	F	Adult	Proto-Three Kingdom (BC 80- 394 AD)
SID-31	Joyeung EⅢ-3ho-3	*Homo sapiens*	Rib	Double Wooden chamber	Retainer	M	21-35	Proto-Three Kingdom (BC 80- 394 AD)
SID-33	Joyeung EⅢ-4ho-2	*Homo sapiens*	Humerus	Double Wooden chamber	Retainer	F	31-50	Proto-Three Kingdom (BC 80- 394 AD)
SID-36	Joyeung EⅢ-8ho-1	*Homo sapiens*	Humerus	Double Wooden chamber	Elite	NA	3-5	Proto-Three Kingdom (BC 80- 394 AD)
SID-37	Joyeung EⅢ-8ho-2	*Homo sapiens*	Humerus	Double Wooden chamber	Retainer	NA	15	Proto-Three Kingdom (BC 80- 394 AD)
SID-46	Joyeung EⅢ-26ho-1	*Homo sapiens*	Phalange	Single Wooden chamber	NA	NA	21-35	Proto-Three Kingdom (BC 80- 394 AD)
SID-47	Joyeung EⅢ-29ho-1	*Homo sapiens*	Rib	Single Wooden chamber	NA	M	Adult	Proto-Three Kingdom (BC 80- 394 AD)

B. $\delta^{13}C$ values of amino acids

Sample ID	Common Name	Bulk	Ala	Asx	Glx	Gly	Hyp	Ile	Leu	Lys	Phe	Pro	Ser	Thr	Val
ERI-HW1	Mumun human	-9.9	-10	-9.1	-8.9	-7.3	-10.4	-21.7	-12.6	-17.4	-18.9	-9.8	0.0	-4.3	-18.8
ERI-HW3	Mumun human	-12.0	-11	-10.7	-9.8	-8	-12.5	-23.3	-12.7	-16.9	-19.9	-10.9	-2.4	-6.3	-20.1
ERICA-KO8	Mumun human	-12.0	-13.8	-12.4	-9.9	-8.7	-12.9	-24.4	-11.7	-18.7	-22.9	-13.2	-4.7	-6.7	-22.1
ERICA-JE9	Mumun human	-11.1	-10.5	-12.7	-8.7	-7.9	-11.9	-23.0	-10.9	-14.7	-19.6	-11.6	-4.3	-5.8	-21.1
SID-04	Imdang human	-18.0	-20.5	-15.6	-16.3	-13.0	-18.9	-21.1	-28.6	-19.2	-26.5	-19.2	-8.8	-9.1	-24.8
SID-05	Imdang human	-18.3	-24.6	-19.6	-18.0	-14.7	-18.7	-21.7	-29.8	-19.0	-28.0	-19.3	-11.4	-8.8	-24.6
SID-07	Imdang human	-18.0	-22.4	-18.0	-18.9	-13.8	-18.4	-19.5	-27.3	-18.2	-28.0	-19.0	-9.2	-6.1	-23.2
SID-08	Imdang human	-17.6	-20.9	-18.8	-16.4	-15.3	-19.4	-20.4	-28.0	-20.0	-27.3	-19.3	-9.6	-9.6	-24.9
SID-09	Imdang human	-19.5	-20.1	-19.5	-16.7	-13.2	-18.6	-21.7	-29.1	-19.3	-26.8	-19.6	-8.4	-3.2	-25.4
SID-10	Imdang human	-17.5	-23.3	-18.3	-17.7	-12.7	-18.6	-21.9	-28.4	-17.9	-27.4	-19.3	-7.7	-1.0	-24.9
SID-11	Imdang human	-18.5	-19.9	-17.5	-17.3	-14.0	-18.8	-21.1	-28.1	-17.5	-27.4	-19.5	-10.1	-2.9	-25.5
SID-12	Imdang human	-20.2	-25.4	-20.9	-19.9	-16.0	-19.9	-25.2	-32.4	-19.6	-29.3	-21.3	-10.8	-4.8	-26.5
SID-13	Imdang human	-18.0	-20.3	-16.5	-16.4	-13.4	-18.1	-23.0	-27.7	-18.8	-26.6	-19.0	-8.8	-5.7	-24.9
SID-15	Imdang human	-19.0	-20.6	-16.0	-16.2	-13.7	-18.1	-23.9	-29.6	-16.7	-27.6	-19.5	-8.1	-5.6	-25.1
SID-16	Imdang human	-18.4	-18.6	-14.5	-14.6	-12.0	-18.1	-23.8	-29.2	-21.1	-27.0	-18.6	-6.0	-5.2	-24.4
SID-17	Imdang human	-16.8	-15.3	-13.1	-12.9	-10.7	-16.6	-22.2	-26.7	-19.1	-24.9	-17.1	-2.9	-2.2	-23.8
SID-19	Imdang human	-18.8	-22.5	-18.8	-18.9	-16.3	-19.1	-23.7	-29.6	-17.0	-28.2	-19.4	-10.6	-5.9	-25.3
SID-20	Imdang human	-19.6	-20.9	-19.4	-19.5	-15.0	-19.4	-23.4	-30.0	-17.9	-28.4	-20.4	-12.0	-3.5	-25.8

SID-21	Imdang human	-18.9	-20.0	-18.2	-18.2	-14.3	-19.1	-23.0	-29.3	-19.0	-27.6	-20.4	-8.0	-1.2	-25.2
SID-22	Imdang human	-19.8	-22.3	-19.4	-20.6	-13.7	-19.2	-22.5	-29.3	-18.6	-28.5	-20.1	-8.1	-0.7	-24.8
SID-25	Imdang human	-17.7	-21.1	-17.7	-19.2	-14.9	-18.6	-23.3	-28.9	-18.8	-27.0	-19.5	-8.3	-2.5	-25.3
SID-26	Imdang human	-17.3	-18.4	-16.7	-17.1	-13.2	-18.3	-22.8	-28.8	-18.6	-26.7	-18.2	-9.3	-1.6	-24.8
SID-27	Imdang human	-18.0	-22.1	-17.5	-18.7	-15.5	-18.8	-23.7	-30.3	-19.6	-27.3	-20.0	-10.0	-2.3	-25.5
SID-28	Imdang human	-17.4	-21.6	-20.0	-18.4	-13.6	-18.6	-24.4	-29.6	-18.0	-27.0	-19.3	-11.6	-3.9	-26.7
SID-29	Imdang human	-18.6	-23.9	-23.2	-19.7	-15.7	-18.8	-25.2	-30.1	-17.2	-28.0	-19.5	-12.0	-3.5	-25.9
SID-30	Imdang human	-18.7	-23.6	-22.0	-21.4	-15.3	-19.6	-25.7	-30.9	-17.6	-28.3	-20.0	-11.8	-3.1	-26.7
SID-31	Imdang human	-17.8	-19.9	-18.1	-18.3	-12.6	-18.1	-25.2	-28.6	-18.5	-26.7	-18.4	-7.3	-1.4	-26.5
SID-33	Imdang human	-17.0	-19.1	-17.4	-18.8	-14.7	-18.0	-24.8	-29.0	-20.8	-25.9	-18.6	-9.9	-2.9	-26.3
SID-36	Imdang human	-18.1	-20.3	-18.2	-18.0	-12.7	-18.3	-26.7	-28.1	-18.4	-26.4	-18.9	-7.1	-3.9	-25.8
SID-37	Imdang human	-17.8	-20.8	-18.5	-19.1	-13.7	-18.5	-26.6	-28.3	-18.0	-26.7	-19.1	-8.8	-4.2	-25.2
SID-46	Imdang human	-19.7	-22.5	-21.9	-20.3	-15.3	-20.6	-28.4	-30.4	-22.0	-28.2	-20.5	-11.4	-5.3	-26.9
SID-47	Imdang human	-19.9	-23.8	-21.4	-21.1	-16.3	-19.8	-28.5	-30.5	-18.6	-28.8	-20.8	-11.1	-7.4	-27.4

C. $\delta^{15}N$ values of amino acids

Sample ID	Common Name	Bulk	Ala	Gly	Val	Leu	Ile	Pro	Ser	Glx	Phe	Hyp
ERI-HW1	Mumun human	6.9	10.3	6.1	14.0	7.1	6.9	13.0	1.3	13.5	5.6	13.1
ERI-HW3	Mumun human	7.6	11.3	8.8	12.2	8.0	8.0	14.5	3.5	13.3	8.0	14.3
ERICA-KO8	Mumun human	7.4	9.0	8.4	13.9	10.0	9.8	14.9	3.0	13.8	7.5	14.7
ERICA-JE9	Mumun human	6.3	8.7	5.3	11.4	7.9	7.1	12.6	-2.1	11.3	5.3	12.3
SID-04	Imdang human	7.8	10.0	7.4	14.3	9.1	11.8	13.9	2.3	13.8	8.7	13.8
SID-05	Imdang human	13.2	13.9	12.9	18.7	12.1	16.2	20.3	7.0	17.9	10.2	20.4
SID-07	Imdang human	13.2	16.0	13.5	19.8	16.0	17.2	20.2	11.2	18.6	12.6	19.9
SID-08	Imdang human	10.0	12.9	9.1	17.3	12.2	15.5	17.5	5.7	15.9	9.9	17.2
SID-09	Imdang human	9.6	16.1	7.8	14.3	15.4	18.7	17.4	4.0	15.8	9.1	17.6
SID-10	Imdang human	10.1	12.3	9.1	17.2	11.5	11.8	18.9	6.0	16.6	10.9	19.1
SID-11	Imdang human	9.9	12.1	8.3	16.3	12.7	14.3	18.2	5.7	16.0	8.9	18.5
SID-12	Imdang human	12.0	14.5	11.4	18.9	13.7	14.1	19.6	5.5	18.2	11.0	19.6
SID-13	Imdang human	9.2	12.3	8.8	16.7	11.7	13.5	17.2	5.9	15.5	9.1	17.4

SID-15	Imdang human	9.8	14.6	10.0	18.1	13.0	10.8	18.7	7.1	16.8	8.7	18.1
SID-16	Imdang human	8.0	11.0	8.0	15.4	9.4	13.2	16.7	5.1	15.1	9.4	16.3
SID-17	Imdang human	7.6	10.6	7.1	14.2	9.3	12.1	15.7	6.9	13.7	10.4	16.1
SID-19	Imdang human	12.3	15.7	13.3	18.6	14.3	16.7	21.4	5.3	18.9	11.3	21.1
SID-20	Imdang human	12.0	16.2	12.9	17.9	12.6	15.2	21.6	4.6	19.0	9.8	21.3
SID-21	Imdang human	10.7	14.7	11.7	17.7	12.0	13.6	19.4	3.9	19.3	12.6	19.6
SID-22	Imdang human	12.4	17.3	13.0	20.6	12.8	13.7	22.2	6.7	20.9	13.4	21.9
SID-25	Imdang human	10.1	15.1	10.5	17.7	9.9	12.5	18.8	2.9	18.6	10.8	18.3
SID-26	Imdang human	8.1	9.7	5.9	11.4	7.4	8.2	13.5	-0.2	13.4	7.7	13.3
SID-27	Imdang human	9.6	13.0	9.5	16.4	10.2	11.1	16.5	1.7	14.9	9.2	16.1
SID-28	Imdang human	10.1	12.6	8.1	17.0	12.5	11.5	17.2	2.4	15.4	8.3	17.3
SID-29	Imdang human	12.6	15.7	11.5	19.3	15.0	16.4	20.0	5.3	18.6	9.3	19.6
SID-30	Imdang human	11.8	15.1	9.7	17.0	12.4	13.3	18.2	5.9	17.5	8.8	18.1
SID-31	Imdang human	10.8	14.6	10.3	16.4	10.7	12.1	17.9	7.4	17.4	8.6	17.9
SID-33	Imdang human	9.0	11.5	7.3	12.4	11.4	12.6	15.8	3.0	14.0	9.4	16.3
SID-36	Imdang human	9.8	11.6	8.6	12.9	10.7	12.5	16.3	3.8	14.8	9.1	16.4
SID-37	Imdang human	9.9	13.1	8.7	13.7	11.1	12.2	16.8	4.3	15.4	8.7	16.6
SID-46	Imdang human	11.0	11.8	8.7	14.7	11.4	14.6	16.4	2.7	14.9	9.4	16.6
SID-47	Imdang human	10.4	14.3	9.2	15.7	11.5	13.4	19.3	6.3	17.0	9.9	19.3

Supplementary Table 2. Information (A) and stable carbon (B) and nitrogen (C) values of amino acids and bulk collagen of the animal remains analyzed in this study.

A. Sample Information

Sample ID	Location	Element	Common Name	Species Name	Food Group	Chronology
SID-50	Joyeung EI-1 Ho	Vertebrate	Sandbar Shark	*Carcharhinus*	Marine animals	80 BC-394 AD
SID-51	Joyeung EI-2 Ho	Vertebrate	Sandbar Shark	*Carcharhinus*	Marine animals	80 BC-394 AD
SID-53	Joyeung EII-2 Ho	Vertebrate	Amberjack	*Seriola*	Marine animals	80 BC-394 AD
SID-58	Joyeung EII-3Ho	Femur	Pheasant	*Phasianus colchicus*	Game birds	80 BC-394 AD
SID-60	Joyeung EII-3Ho	Tarsal	Great Bustard	*Otis tarda*	Game birds	80 BC-394 AD
SID-61	Joyeung EII-3 Ho	Humerus	Wild goose	*Anser fabalis*	Game birds	80 BC-394 AD
SID-63	Joyeung EII-4 Ho	Vertebrate	Swan	*Cygnus columbianus*	Game birds	80 BC-394 AD
SID-65	Joyeung EII-5 Ho	Metacarpal	Cattle	*Bos taurus*	Terrestrial herbivores	80 BC-394 AD
SID-68	Imdang 2 Ho North	Phalange	Wild boar	*Sus scrofa*	Terrestrial herbivores	80 BC-394 AD
ERICA-JE14	Jeongseon MaeDun	Tibia	Wild boar	*Sus Scrofa*	Terrestrial herbivores	566 BC
ERICA-JE11	Jeongseon MaeDun	Tibia	Deer	*Cervus Nippon*	Terrestrial herbivores	566 BC
SID-70	Imdang 6A-ho	Tooth	Pig	*Sus domesticus*	Terrestrial herbivores	80 BC-394 AD
SID-71	Imdang 2 Ho North	Vertebrate	Hare	*Lepus*	Terrestrial herbivores	80 BC-394 AD

B. $\delta^{13}C$ values of amino acids

Sample ID	Common Name	bulk	Ala	Asx	Glx	Gly	Hyp	Ile	Leu	Lys	Phe	Pro	Ser	Thr	Val
SID-50	Sandbar Shark	-12.5	-17.1	-14.4	-15.1	-2.1	-14.9	-20.6	-23.6	-15.5	-24.7	-15.0	0.4	1.6	-20.0
SID-51	Sandbar Shark	-12.6	-15.6	-13.5	-13.3	-3.5	-15.1	-19.2	-22.9	-13.6	-24.3	-15.1	1.8	1.4	-19.0
SID-53	Amberjack	-12.0	-16.1	-13.3	-14.0	-2.0	-13.8	-20.4	-22.9	-16.0	-24.0	-14.8	-0.5	-0.7	-19.0
SID-58	Pheasant	-16.2	-23.0	-18.1	-19.1	-14.2	-19.5	-25.5	-29.2	-22.4	-26.4	-19.5	-11.4	-5.5	-25.5
SID-60	Great Bustard	-18.5	-25.5	-19.8	-20.0	-15.8	-18.6	-23.6	-29.7	-19.8	-25.4	-19.8	-14.3	-6.0	-24.2
SID-61	Wild goose	-22.4	-26.6	-24.6	-24.9	-17.5	-22.6	-27.8	-33.5	-24.3	-29.3	-22.8	-17.8	-14.5	-28.9
SID-63	Swan	-15.3	-16.7	-14.8	-14.8	-12.6	-15.3	-23.3	-26.3	-17.7	-22.8	-15.4	-8.7	-11.3	-21.7
SID-65	Cattle	-14.0	-17.5	-10.6	-10.3	-8.6	-13.0	-20.3	-23.8	-15.0	-21.9	-13.4	-2.3	-5.4	-21.6
SID-68	Wild boar	-20.4	-21.7	-16.0	-19.0	-16.2	-19.0	-22.4	-28.9	-21.8	-26.9	-19.5	-9.5	-8.2	-25.7
ERICA-JE14	Wild boar	-19.6	-24.3	-21.8	-21.3	-18.8	-18.5	-30.5	-20.7	-21.7	-28.4	-19.9	-15.6	-11.5	-29.0

ERICA-JE11	Deer	-21.5	-27.0	-20.7	-21.1	-18.2	-19.8	-32.0	-23.5	-19.7	-27.8	-20.8	-15.0	-16.7	-28.8
SID-70	Pig	-19.4	-21.3	-16.0	-17.8	-11.9	-18.1	-25.0	-28.3	-21.0	-26.2	-18.6	-10.7	-9.5	-25.7
SID-71	Hare	-21.1	-22.9	-19.2	-19.6	-15.9	-21.5	-27.4	-31.8	-21.5	-27.4	-21.5	-12.1	-10.1	-28.9

C. $\delta^{15}N$ values of amino acids

Sample ID	Common Name	Bulk	Ala	Gly	Val	Leu	Ile	Pro	Ser	Glx	Phe	Hyp
SID-50	Sandbar Shark	11.9	24.5	3.7	29.6	25.9	28.2	23.4	1.9	27.5	8.6	22.9
SID-51	Sandbar Shark	13.0	25.2	3.6	30.1	27.0	32.0	24.1	4.2	29.2	8.0	23.6
SID-53	Amberjack	12.1	24.9	5.4	28.3	24.9	29.6	23.6	5.0	27.6	7.5	23.6
SID-58	Pheasant	5.2	7.4	5.1	9.7	4.9	6.7	12.4	5.1	10.5	8.5	12.6
SID-60	Great Bustard	5.9	8.3	6.7	8.9	10.5	10.7	14.9	2.6	10.6	8.6	15.2
SID-61	Wild goose	9.8	12.4	8.9	15.7	10.8	9.0	14.2	2.7	15.3	17.1	14.4
SID-63	Swan	7.2	10.3	8.4	13.5	8.0	9.1	15.9	4.5	11.9	11.1	15.7
SID-65	Cattle	6.6	8.5	7.7	11.8	6.3	4.3	12.2	5.6	10.8	10.2	12.5
SID-68	Wild boar	2.3	4.2	1.9	9.1	4.5	5.7	8.3	3.6	8.3	8.3	8.2
ERICA-JE14	Wild boar	3.3	4.4	2.1	9.0	6.2	5.9	9.0	-2.3	8.5	6.9	9.1
ERICA-JE11	deer	3.7	5.0	3.2	9.5	7.7	6.7	9.1	2.0	8.6	9.0	9.7
SID-70	Pig	7.2	8.1	6.4	11.9	7.7	7.1	11.7	3.1	11.2	9.4	11.0
SID-71	Hare	3.1	3.2	2.0	10.6	4.8	6.1	9.2	0.2	9.2	9.1	8.9

Supplementary Table 3. Information (A) and stable carbon (B) and nitrogen (C) values of amino acids and bulk collagen of the cereals analyzed in this study

A. Sample Information

Sample ID	Location	Common Name	Species Name	Food Group	Chronology
SID-102	South Korea	Sorghum	*Sorghum bicolor*	C4 plants	Modern
SID-103	South Korea	Common millet	*Panicum miliaceum*	C4 plants	Modern
SID-104	South Korea	Foxtail millet	*Setaria italica*	C4 plants	Modern
SID-105	South Korea	Rice	*Oryza sativa*	C3 plants	Modern
SID-106	South Korea	Soybean	*Glycine max*	C3 plants	Modern
SID-107	South Korea	Azuki bean	*Vigna angularis*	C3 plants	Modern
SID-108	South Korea	Wheat	*Triticum aestivum*	C3 plants	Modern
SID-109	South Korea	Barley	*Hordeum vulgare*	C3 plants	Modern
SID-101	Primorsky Krai, Russia	Oat	*Avena sativa*	C3 plants	Modern

B. $\delta^{13}C$ values of amino acids

Sample ID	Common Name	bulk	Ala	Asx	Glx	Gly	Met	Ile	Leu	Tyr	Phe	Pro	Ser	Thr	Val
SID-102	Sorghum	-11.4	-10.3	-6.4	-11.7	-1.7	-16.5	-13.9	-16.3	-11.7	-13.9	-12.1	-5.8	-11.1	-21.3
SID-103	Common millet	-11.8	-11.7	-9.8	-10.0	-1.3	-15.1	-14.6	-16.3	-11.7	-13.9	-12.6	-5.7	-9.5	-23.4
SID-104	Fox millet	-12.1	-13.0	-7.9	-12.7	-1.1	-17.3	-16.0	-19.5	-12.9	-14.9	-12.5	-5.6	-10.3	-22.6
SID-105	Rice	-27.4	-25.9	-25.7	-26.1	-15.5	-27.3	-28.1	-29.6	-25.3	-27.7	-24.0	-11.0	-19.5	-36.9
SID-106	Soybean	-27.2	-22.5	-17.8	-24.0	-14.7	-30.2	-26.9	-29.0	-27.9	-27.9	-24.9	-11.6	-18.5	-36.2
SID-107	Azuki bean	-26.2	-26.7	-21.5	-25.3	-16.9	-30.8	-28.5	-31.3	-28.6	-29.4	-25.1	-13.8	-20.4	-36.0
SID-108	Wheat	-27.3	-29.9	-25.5	-27.1	-15.3	-33.1	-29.8	-32.1	-25.0	-30.0	-27.2	-12.7	-16.9	-34.7
SID-109	Barley	-27.0	-41.4	-27.7	-28.1	-23.0	-34.1	-30.4	-32.8	-26.7	-29.4	-28.7	-16.0	-16.6	-35.6
SID-101	Oat	-28.5	-29.3	-27.0	-23.6	-12.6	-30.7	-28.7	-31.3	-23.0	-28.4	-25.8	-11.6	-12.6	-35.8

C. δ^{15}N values of amino acids

Sample ID	Common Name	bulk	Ala	Gly	Val	Leu	Ile	Pro	Ser	Glx	Phe
SID-102	Sorghum	2.9	1.9	1.6	4.2	-0.2	1.2	4.2	-4.5	2.2	3.1
SID-103	Common millet	3.0	5.0	1.6	5.4	2.2	3.2	7.1	-4.5	6.0	6.0
SID-104	Fox millet	3.2	3.9	4.9	6.6	2.4	4.8	6.3	-4.1	5.4	8.4
SID-105	Rice	4.5	5.2	3.3	6.3	3.2	4.5	7.8	-3.3	4.8	6.6
SID-106	Soybean	4.0	4.9	2.9	7.8	2.5	4.3	10.8	-3.3	5.4	8.3
SID-107	Azuki bean	3.6	2.0	-0.8	2.3	-1.4	0.7	6.0	-6.2	2.6	3.3
SID-108	Wheat	2.8	9.9	8.7	11.2	8.5	9.1	13.8	5.8	11.2	13.1
SID-109	Barley	2.6	4.5	5.7	5.5	5.3	6.4	6.3	1.0	6.8	15.0
SID-101	Oat	2.9	3.5	2.2	5.6	-0.4	1.8	6.3	-4.4	6.0	9.3

Supplementary Table 4. Principal component analysis outfit of 9 essential amino acids from food sources and humans.

Vector scores

	PC1	PC2	PC3
Ile_C	-0.34	0.33	0.22
Leu_C	-0.29	0.37	-0.12
Phe_C	-0.27	0.44	0.29
Thr_C	-0.42	-0.07	0.03
Val_C	-0.43	0.12	0.19
Val_N	-0.35	-0.37	-0.08
Leu_N	-0.34	-0.39	-0.11
Ile_N	-0.33	-0.38	-0.17
Phe_N	0.13	-0.38	0.83
Proportion (%)	50	32	7

Sample scores

ID		PC1	PC2	PC3
SID-50	Sandbar Shark	-4.08	-1.95	-0.51
SID-51	Sandbar Shark	-4.52	-1.91	-0.68
SID-53	Amberjack	-4.07	-1.60	-0.75
SID-58	pheasant	0.13	-0.10	0.04
SID-60	Great Bustard	-0.44	-0.28	0.10
SID-61	Wild goose	1.14	-2.36	1.80
SID-63	Swan	-0.56	0.02	0.94
SID-65	Cattle	-1.02	0.83	1.01
SID-68	Wild boar	0.20	0.22	0.09
ERICA-JE14	Pig	0.10	-0.27	0.19
ERICA-JE11	Hare	0.99	-0.56	-0.03
SID-70	Deer	1.16	-0.29	-0.53
SID-71	Wild boar	0.66	0.23	-1.05
SID-101	Oat	2.35	-0.16	-0.14
SID-102	Rice	2.33	0.17	-1.05
SID-103	Soybean	2.14	0.03	-0.54
SID-104	Azuki bean	2.97	0.81	-1.79
SID-105	Wheat	1.95	-1.74	0.41
SID-106	Barley	2.60	-1.50	1.09
SID-107	Sorghum	-1.39	4.19	-0.12
SID-108	Common millet	-1.45	3.51	0.47
SID-109	Fox millet	-1.19	2.72	1.04

Sample scores

ID	Species	PC1	PC2	PC3
SID-04	Imdang Human	-0.11	0.41	-0.04
SID-05	Imdang Human	-0.88	-0.67	-0.19
SID-07	Imdang Human	-1.93	-0.68	-1.07
SID-08	Imdang Human	-0.82	-0.10	-0.21
SID-09	Imdang Human	-1.34	-0.35	0.30
SID-10	Imdang Human	-1.05	-0.39	-0.62
SID-11	Imdang Human	-1.07	-0.07	0.19
SID-12	Imdang Human	-0.61	-1.81	-0.15
SID-13	Imdang Human	-0.72	-0.01	0.15
SID-15	Imdang Human	-0.56	-0.47	0.35
SID-16	Imdang Human	-0.41	-0.23	-0.03
SID-17	Imdang Human	-0.80	0.51	-0.66
SID-19	Imdang Human	-1.08	-1.20	-0.32
SID-20	Imdang Human	-0.92	-0.93	0.12
SID-21	Imdang Human	-1.10	-1.02	-1.06
SID-22	Imdang Human	-1.48	-1.36	-1.29
SID-25	Imdang Human	-0.75	-0.51	-0.51
SID-26	Imdang Human	0.05	0.57	0.24
SID-27	Imdang Human	-0.47	-0.46	0.04
SID-28	Imdang Human	-0.43	-0.42	0.56
SID-29	Imdang Human	-1.13	-1.17	0.54
SID-30	Imdang Human	-0.44	-1.05	0.58
SID-31	Imdang Human	-0.49	-0.31	0.47
SID-33	Imdang Human	-0.25	-0.19	0.07
SID-36	Imdang Human	-0.08	-0.25	0.33
SID-37	Imdang Human	-0.17	-0.25	0.47
SID-46	Imdang Human	0.06	-1.24	0.57
SID-47	Imdang Human	0.29	-1.45	0.43
ERI-HW1	Mumun Human	-1.96	1.65	-0.33
ERI-HW3	Mumun Human	-1.46	1.15	0.10
ERICA-KO8	Mumun Human	-1.36	0.62	-0.47
ERICA-JE9	Mumun Human	-1.57	1.69	-0.64

Supplementary Table 5. Isotopic data of food sources (A) and trophic discrimination factors (B) of six EAAs used for the MixSIAR model (bulk and EAA isotope model).

I. Bulk isotope model

A. Food sources

Source	Mean $\delta^{13}C$	SD $\delta^{13}C$	Mean $\delta^{15}N$	SD $\delta^{15}N$	n
C4_plants	-11.8	0.4	3	0.2	3
Game birds	-17.6	3.1	7	1.6	4
Terrestrial_herbivores	-20.7	0.8	3.1	0.6	4
Marine	-12.4	0.3	12.3	0.6	3
C3_plants	-27.3	0.7	3.4	0.8	6

B. Trophic discrimination factors

Source	Mean $\delta^{13}C$	SD $\delta^{13}C$	Mean $\delta^{15}N$	SD $\delta^{15}N$
C4_plants	4.4	0.15	3.8	0.74
Game birds	1	0.63	3.8	0.74
Terrestrial_herbivores	1	0.63	3.8	0.74
Marine	1	0.63	3.8	0.74
C3_plants	5.2	0.23	3.8	0.74

II. EAA isotope model

A. Food sources

Source	MeanThr_C	SDThr_C	MeanVal_C	SDVal_C	MeanIle_C	SDIle_C	MeanLeu_C	SDLeu_C	MeanPhe_C	SDPhe_C	MeanPhe_N	SDPhe_N	n
Marine	0.8	1.3	-19.3	0.6	-20.1	0.8	-23.1	0.4	-24.4	0.3	8.1	0.5	3
Game birds	-9.3	4.3	-25.1	3	-25.1	2.1	-29.6	2.9	-26	2.7	11.3	4	4
Terrestrial Herbivores	-11.6	3.6	-28.1	1.6	-28.1	4.2	-26.2	5	-27.6	0.6	8.3	1	4
C4_plants	-10.3	0.8	-22.4	1	-14.8	1.1	-17.4	1.8	-14.2	0.6	5.8	2.7	3
C3_plants	-17.4	2.8	-35.9	0.8	-28.7	1.2	-31	1.5	-28.8	0.9	9.3	4.3	6

B. Trophic discrimination factors

Source	MeanThr_C	SDThr_C	MeanVal_C	SDVal_C	MeanIle_C	SDIle_C	MeanLeu_C	SDLeu_C	MeanPhe_C	SDPhe_C	MeanPhe_N	SDPhe_N
Marine	0	0	0	0	0	0	0	0	0	0	0	0
Game birds	0	0	0	0	0	0	0	0	0	0	0	0
Terrestrial_herbivores	0	0	0	0	0	0	0	0	0	0	0	0
C4_plants	0	0	0	0	0	0	0	0	0	0	0	0
C3_plants	0	0	0	0	0	0	0	0	0	0	0	0

Supplementary Table 6. MixSIAR model estimated results for each human in proportional contribution of five food sources with Bayesian Credibility Intervals (5% and 95%).

I. Bulk isotope model

		**C3 plants**			**Terrestrial herbivores**			**Game birds**			**Marine animals**			**C4 plants**		
**Sample ID**	**Common Name**	Mean %	5% Cl	95% Cl	Mean %	5% Cl	95% Cl	Mean %	5% Cl	95% Cl	Mean %	5% Cl	95% Cl	Mean %	5% Cl	95% Cl
ERI-HW1	Mumun human	0.06	0.00	0.14	0.08	0.01	0.18	0.09	0.01	0.24	0.04	0.00	0.11	0.73	0.59	0.82
ERI-HW3	Mumun human	0.11	0.01	0.25	0.14	0.01	0.30	0.13	0.01	0.37	0.07	0.01	0.16	0.55	0.38	0.66
ERICA-KO8	Mumun human	0.12	0.01	0.25	0.15	0.01	0.32	0.12	0.01	0.35	0.06	0.01	0.14	0.56	0.38	0.66
ERICA-JE9	Mumun human	0.09	0.01	0.22	0.12	0.01	0.27	0.11	0.00	0.39	0.03	0.00	0.09	0.65	0.41	0.75
SID-04	Imdang human	0.31	0.03	0.61	0.33	0.03	0.69	0.16	0.01	0.44	0.07	0.01	0.17	0.16	0.01	0.24
SID-05	Imdang human	0.13	0.01	0.36	0.10	0.01	0.28	0.50	0.23	0.76	0.21	0.02	0.47	0.05	0.00	0.16
SID-07	Imdang human	0.13	0.01	0.34	0.11	0.01	0.29	0.49	0.23	0.74	0.22	0.02	0.46	0.06	0.00	0.16
SID-08	Imdang human	0.27	0.03	0.53	0.22	0.02	0.52	0.24	0.02	0.56	0.19	0.03	0.33	0.08	0.01	0.19
SID-09	Imdang human	0.36	0.04	0.67	0.20	0.01	0.52	0.27	0.03	0.60	0.11	0.01	0.24	0.05	0.00	0.15
SID-10	Imdang human	0.23	0.03	0.51	0.23	0.02	0.52	0.24	0.02	0.56	0.20	0.04	0.35	0.08	0.01	0.19
SID-11	Imdang human	0.31	0.03	0.59	0.22	0.02	0.54	0.26	0.02	0.57	0.16	0.02	0.29	0.06	0.00	0.17
SID-12	Imdang human	0.16	0.01	0.41	0.12	0.01	0.31	0.52	0.26	0.77	0.15	0.01	0.35	0.06	0.00	0.16
SID-13	Imdang human	0.29	0.03	0.58	0.28	0.02	0.66	0.21	0.02	0.51	0.14	0.02	0.27	0.08	0.01	0.20
SID-15	Imdang human	0.34	0.03	0.64	0.21	0.02	0.53	0.26	0.02	0.58	0.14	0.02	0.26	0.06	0.00	0.17
SID-16	Imdang human	0.33	0.04	0.63	0.33	0.03	0.70	0.16	0.01	0.42	0.08	0.01	0.17	0.10	0.01	0.21
SID-17	Imdang human	0.25	0.02	0.54	0.32	0.03	0.65	0.16	0.01	0.45	0.07	0.01	0.17	0.20	0.05	0.32
SID-19	Imdang human	0.17	0.01	0.43	0.12	0.01	0.32	0.48	0.20	0.74	0.19	0.02	0.39	0.06	0.00	0.15
SID-20	Imdang human	0.17	0.01	0.45	0.12	0.01	0.33	0.51	0.23	0.77	0.15	0.01	0.36	0.06	0.00	0.16

SID-21	Imdang human	0.27	0.03	0.59	0.17	0.01	0.44	0.34	0.04	0.66	0.16	0.02	0.31	0.06	0.00	0.16
SID-22	Imdang human	0.15	0.01	0.39	0.12	0.01	0.30	0.52	0.26	0.77	0.16	0.01	0.38	0.05	0.00	0.15
SID-25	Imdang human	0.27	0.03	0.54	0.22	0.02	0.53	0.24	0.02	0.56	0.19	0.04	0.34	0.07	0.03	0.54
SID-26	Imdang human	0.27	0.03	0.55	0.32	0.04	0.65	0.17	0.01	0.44	0.09	0.01	0.20	0.15	0.03	0.28
SID-27	Imdang human	0.29	0.03	0.56	0.25	0.02	0.55	0.23	0.02	0.54	0.16	0.03	0.30	0.08	0.01	0.18
SID-28	Imdang human	0.25	0.03	0.51	0.23	0.02	0.51	0.24	0.02	0.55	0.21	0.04	0.35	0.08	0.01	0.19
SID-29	Imdang human	0.15	0.01	0.38	0.12	0.01	0.32	0.49	0.21	0.76	0.19	0.02	0.41	0.06	0.00	0.16
SID-30	Imdang human	0.19	0.01	0.49	0.13	0.01	0.35	0.44	0.13	0.72	0.19	0.02	0.38	0.06	0.00	0.16
SID-31	Imdang human	0.25	0.02	0.53	0.18	0.15	0.46	0.29	0.02	0.62	0.21	0.04	0.37	0.06	0.00	0.17
SID-33	Imdang human	0.25	0.02	0.50	0.28	0.03	0.59	0.21	0.02	0.50	0.14	0.02	0.27	0.13	0.02	0.25
SID-36	Imdang human	0.30	0.03	0.57	0.23	0.02	0.56	0.23	0.01	0.55	0.17	0.03	0.31	0.07	0.01	0.18
SID-37	Imdang human	0.29	0.04	0.55	0.22	0.02	0.52	0.24	0.02	0.56	0.19	0.03	0.33	0.07	0.01	0.19
SID-46	Imdang human	0.24	0.02	0.59	0.15	0.01	0.38	0.43	0.11	0.71	0.14	0.01	0.29	0.06	0.00	0.20
SID-47	Imdang human	0.28	0.02	0.64	0.16	0.01	0.43	0.38	0.08	0.68	0.12	0.01	0.26	0.06	0.00	0.16

II. EAA isotope model

		C_3 plants			Terrestrial herbivores			Game birds			Marine animals			C_4 plants		
**Sample ID**	**Common Name**	Mean %	5% Cl	95% Cl	Mean %	5% Cl	95% Cl	Mean %	5% Cl	95% Cl	Mean %	5% Cl	95% Cl	Mean %	5% Cl	95 % Cl
ERI-HW1	Mumun human	0.03	0.00	0.07	0.06	0.00	0.26	0.39	0.01	0.71	0.15	0.01	0.42	0.37	0.09	0.62
ERI-HW3	Mumun human	0.03	0.00	0.09	0.10	0.00	0.31	0.39	0.02	0.72	0.18	0.01	0.42	0.31	0.04	0.54
ERICA-KO8	Mumun human	0.03	0.00	0.09	0.29	0.03	0.50	0.17	0.01	0.60	0.27	0.05	0.46	0.24	0.08	0.33
ERICA-JE9	Mumun human	0.03	0.00	0.08	0.08	0.00	0.28	0.27	0.01	0.68	0.23	0.02	0.44	0.39	0.08	0.57
SID-04	Imdang human	0.09	0.01	0.21	0.34	0.03	0.64	0.26	0.02	0.59	0.24	0.05	0.41	0.06	0.00	0.19

SID-05	Imdang human	0.09	0.01	0.22	0.19	0.01	0.50	0.49	0.24	0.74	0.16	0.02	0.34	0.07	0.00	0.19
SID-07	Imdang human	0.06	0.00	0.15	0.10	0.01	0.29	0.54	0.34	0.76	0.23	0.03	0.44	0.08	0.01	0.22
SID-08	Imdang human	0.09	0.01	0.22	0.27	0.02	0.62	0.39	0.10	0.66	0.17	0.02	0.34	0.08	0.01	0.21
SID-09	Imdang human	0.08	0.01	0.20	0.27	0.01	0.63	0.34	0.03	0.68	0.26	0.05	0.45	0.05	0.00	0.20
SID-10	Imdang human	0.07	0.01	0.18	0.12	0.01	0.38	0.51	0.29	0.74	0.24	0.04	0.44	0.06	0.00	0.16
SID-11	Imdang human	0.08	0.01	0.20	0.23	0.01	0.64	0.40	0.06	0.69	0.24	0.04	0.45	0.05	0.00	0.16
SID-12	Imdang human	0.12	0.01	0.28	0.15	0.01	0.41	0.59	0.35	0.81	0.10	0.01	0.24	0.04	0.00	0.10
SID-13	Imdang human	0.14	0.02	0.28	0.19	0.01	0.49	0.26	0.04	0.52	0.37	0.15	0.53	0.04	0.00	0.12
SID-15	Imdang human	0.11	0.01	0.26	0.22	0.01	0.55	0.40	0.12	0.69	0.23	0.04	0.41	0.04	0.00	0.13
SID-16	Imdang human	0.10	0.01	0.24	0.20	0.01	0.48	0.38	0.11	0.66	0.28	0.07	0.46	0.04	0.00	0.12
SID-17	Imdang human	0.09	0.01	0.20	0.10	0.01	0.26	0.29	0.09	0.53	0.46	0.22	0.63	0.06	0.00	0.18
SID-19	Imdang human	0.12	0.01	0.26	0.14	0.01	0.36	0.51	0.29	0.74	0.19	0.03	0.36	0.05	0.00	0.13
SID-20	Imdang human	0.10	0.01	0.23	0.16	0.01	0.46	0.53	0.27	0.77	0.17	0.02	0.35	0.05	0.00	0.13
SID-21	Imdang human	0.09	0.01	0.22	0.10	0.01	0.29	0.58	0.38	0.79	0.18	0.02	0.37	0.05	0.00	0.14
SID-22	Imdang human	0.07	0.01	0.18	0.09	0.01	0.25	0.63	0.44	0.82	0.16	0.02	0.34	0.05	0.00	0.15
SID-25	Imdang human	0.10	0.01	0.24	0.14	0.01	0.41	0.48	0.22	0.72	0.23	0.03	0.43	0.05	0.00	0.14
SID-26	Imdang human	0.08	0.01	0.20	0.23	0.01	0.61	0.36	0.03	0.67	0.28	0.06	0.48	0.05	0.00	0.14
SID-27	Imdang human	0.09	0.01	0.23	0.24	0.01	0.63	0.44	0.07	0.74	0.19	0.03	0.38	0.04	0.00	0.12
SID-28	Imdang human	0.15	0.01	0.33	0.32	0.02	0.71	0.31	0.03	0.65	0.19	0.03	0.37	0.04	0.00	0.11
SID-29	Imdang human	0.12	0.01	0.29	0.21	0.01	0.58	0.48	0.16	0.76	0.15	0.02	0.32	0.04	0.00	0.11
SID-30	Imdang human	0.13	0.01	0.31	0.25	0.02	0.67	0.47	0.11	0.76	0.11	0.01	0.26	0.03	0.00	0.10
SID-31	Imdang human	0.13	0.01	0.31	0.33	0.02	0.70	0.30	0.02	0.65	0.20	0.03	0.38	0.04	0.00	0.11
SID-33	Imdang human	0.13	0.01	0.30	0.27	0.02	0.59	0.32	0.04	0.63	0.23	0.04	0.42	0.05	0.00	0.14
SID-36	Imdang human	0.11	0.01	0.26	0.37	0.07	0.65	0.23	0.02	0.58	0.25	0.05	0.42	0.04	0.00	0.10
SID-37	Imdang human	0.10	0.01	0.24	0.37	0.06	0.66	0.25	0.02	0.61	0.25	0.05	0.43	0.03	0.00	0.10

SID-46	Imdang human	0.12	0.01	0.29	0.39	0.05	0.77	0.36	0.06	0.69	0.09	0.01	0.23	0.03	0.00	0.08
SID-47	Imdang human	0.16	0.02	0.35	0.33	0.04	0.66	0.41	0.12	0.69	0.08	0.01	0.20	0.03	0.00	0.08

1. PCA R code for EAA isotope analysis

The following code was used to PCA analysis of with five $\delta^{13}C$ (Phe, Leu, Val, Ile, Thr) and four $\delta^{15}N$ (Val, Leu, Ile, Phe) values of essential amino acids. This model was run at the R studio.

```
#call libraries ("MASS")
library(MASS)
library(readxl)
library(ggplot2)


#load excel data
exceldata = read_excel("/Users/kyungcheol/Desktop/Humans.xlsx")
Humans = data.frame(exceldata)
Humans


#Run PCA
Humans.pca <- prcomp(humans[, -1],
                center = TRUE, scale=TRUE, retx = T)

# Extract loadings of the variables
PCAloadings <- data.frame(Variables = rownames(humans.pca$rotation),
                     Humans.pca$rotation)

# Plot PCA data
p <- ggplot(humans.pca, aes(x = PC1, y = PC2, color = humans$species))

+geom_point()+stat_ellipse(geom="polygon",alpha=.3,aes(fill=humans$species))

p<p+geom_hline(aes(yintercept=0), linewidth=.4)+geom_vline(aes(xintercept=0),
linewidth=.4)
```

2.MixSIAR R code for bulk isotope Data

The following code was used to calculate dietary contribution estimates for Mumun and Imdang people using bulk carbon and nitrogen stable isotope data. The model takes three .CSV file (sources, humans, trophic discrimination factors) and read it to the MixSIAR model. This model was run at the R studio.

```
library(MixSIAR) ## Call libraries

#loading mixture (human) data
Humans<-read.table("/Users/kyungcheol/Desktop/humans.csv")

#Loading source data
sources<-read.table("/Users/kyungcheol/Desktop/sources.csv")

#Loading discrimination factor data
tdf<-read.table("/Users/kyungcheol/Desktop/tdf.csv")
mix<-load_mix_data (filename="/Users/kyungcheol/Desktop/humans.csv",
              iso_names=c("d13C","d15N"),
              factors="sample",
              fac_random=FALSE,
              fac_nested=FALSE,
              cont_effects=NULL)
source<-load_source_data (filename="/Users/kyungcheol/Desktop/sources.csv",
                  source_factors=NULL,
                  conc_dep=FALSE,
                  data_type="means", mix)
discr<-load_discr_data(filename="/Users/kyungcheol/Desktop/tdf.csv", mix)

#Making an isospace plot
plot_data (filename="isospace_plot", plot_save_pdf=TRUE,
        plot_save_png=FALSE, mix, source, discr)

if(mix$n.iso==2) calc_area(source=source, mix=mix, discr=discr)
```

```
#Plot your prior
plot_prior(alpha.prior=1,source)

#Error structure options
model_filename <- "MixSIAR_model.txt"
resid_err <- FALSE
process_err <- TRUE

#Run MixSIAR (normal)

write_JAGS_model(model_filename, resid_err, process_err, mix, source)

jags.1 <- run_model (run="normal",mix,source, discr, model_filename,
              alpha.prior = 1,resid_err,process_err)

#Check diagnostics and interpreting MixSIAR output

output_JAGS(jags.1, mix, source)
```

3. MixSIAR R code for 6 Amino acid isotope Data (Leu_C, Val_C, Ile_C, Thr_C, Phe_C, Phe_N)

The following code was used to calculate dietary contribution estimates for Mumun and Imdang people using 6 AA stable isotope data. The model takes three .CSV file (sources, humans, trophic discrimination factors) and read it to the MixSIAR model. This model was run at the R studio.

```
library(MixSIAR) ## Call libraries

#loading mixture (human) data
Humans<-read.table("/Users/kyungcheol/Desktop/humans.csv")

#Loading source data
sources<-read.table("/Users/kyungcheol/Desktop/sources.csv")

#Loading discrimination factor data
tdf<-read.table("/Users/kyungcheol/Desktop/tdf.csv")
mix<-load_mix_data (filename="/Users/kyungcheol/Desktop/humans.csv",
            iso_names=c("Thr_C","Val_C","Ile_C","Leu_C","Phe_C","Phe_N"), # add 6
AA data
            factors="id",
            fac_random=FALSE,
            fac_nested=FALSE,
            cont_effects=NULL)
source<-load_source_data (filename="/Users/kyungcheol/Desktop/sources.csv",
                source_factors=NULL,
                conc_dep=FALSE,
                data_type="means", mix)
discr<-load_discr_data(filename="/Users/kyungcheol/Desktop/tdf.csv", mix)

#Making an isospace plot
plot_data (filename="isospace_plot", plot_save_pdf=TRUE,
      plot_save_png=FALSE, mix, source, discr)

if(mix$n.iso==2) calc_area(source=source, mix=mix, discr=discr)
```

```
#Plot your prior
plot_prior(alpha.prior=1,source)

#Error structure options
model_filename <- "MixSIAR_model.txt"
resid_err <- FALSE
process_err <- TRUE

#Run MixSIAR (normal)

write_JAGS_model(model_filename, resid_err, process_err, mix, source)

jags.1 <- run_model (run="normal",mix,source, discr, model_filename,
                alpha.prior = 1,resid_err,process_err)

#Check diagnostics and interpreting MixSIAR output

output_JAGS(jags.1, mix, source)
```
