# Supplementary material for: Enhanced dietary reconstruction of Korean prehistoric populations by combining δ13C and δ15N amino acids of bone collagen
Source: PLoS One. 2024 Mar 27;19(3):e0300068. doi: 10.1371/journal.pone.0300068 (PMC10971582; doi:10.1371/journal.pone.0300068)
Supplement: Attachment — Submitted filename: PLOS ONE Review - M Fontanals-Coll.pdf [file pone.0300068.s003.pdf]

PLOS ONE Review

Enhanced dietary reconstruction of Korean prehistoric populations by combining d 13 C and d 15 N amino acids of bone collagen

Reviewer comments to the editor

This article presents highly valuable data for the dietary reconstruction of Korean prehistoric populations. Compound-Specific Stable Isotope Analysis from Amino Acids (CSIA-AA) offers high-resolution insights into the diets of past populations. This cutting-edge methodology in bioarchaeological analysis is increasingly utilised to address specific questions about food sources and trophic levels in ancient communities.

I would like to suggest minor revisions for this article, and I strongly encourage its publication in your journal.

Reviewer comments to the authors

General comments

Dear authors, your article presents highly valuable data for the dietary reconstruction of Korean prehistoric populations. The use of Compound-Specific Stable Isotope Analysis from Amino Acids (CSIA-AA) distinctly strengthens this study and contributes significant insights to the published pool of CSIA-AA data in archaeology. This, in turn, optimises and enriches prospects for future research in the field of dietary reconstructions.

I extend my congratulations to the authors for conducting such an exemplary study. Additionally, I would like to suggest minor revisions that could potentially further enhance the work for its publication.

Furthermore, I encourage the authors to report the scripts for the PCA and the MixSIAR models. Sharing this information would significantly contribute to advancing the use of CSIA-AA in this field and optimise its interpretation.

Minor revisions

- Page 3 (lines 64-66), to the comment “Amino acids (AAs) can be divided into two groups on the basis of their synthesis and function: essential amino acid (EAA) and non-essential amino acid (NEAA)”:

  I would like to suggest to make clear that this distinction is related to the carbon pathways and that it's different for nitrogen, for which they should rather differentiate between source and trophic amino acids (see e.g. O'Connell (2017) on the nitrogen metabolic pool).

  O'Connell TC. (2017) 'Trophic' and 'source' amino acids in trophic estimation: a likely metabolic explanation. Oecologia. 184(2):317-326. doi: 10.1007/s00442-017-3881-9.

- Page 8 (line 216), about derivatisation method(s?):

  It's important to note that the derivatisation process applies to the actual amino acid, not the isotope value itself. However, I'm unclear on whether you employed two distinct derivatisation approaches—one for measuring nitrogen isotope values and another for carbon. Your description suggests this might be the case, but it's not entirely clear, especially regarding the method used for carbon isotope measurements. Could you please provide clarification on this aspect?

- Page 10 (lines 273 - 274), to the sentence: “Standard deviations of carbon isotope measurements were generally ±0.4‰ as determined by repeated injections of the 10 AA standard mixture”:

Have you considered propagating the error due to inclusion of exogenous carbon from your solvents? I believe that's something important to do. See Docherty et al (2001)

Docherty G, Jones V, Evershed RP. Practical and theoretical considerations in the gas chromatography/combustion/isotope ratio mass spectrometry delta(13)C analysis of small polyfunctional compounds. Rapid Commun Mass Spectrom. 2001;15(9):730-8. doi: 10.1002/rcm.270. PMID: 11319796.

- Page 11 (line 312), regarding: “four essential (Ile, Leu, Phe, Val)”:

  Please, be cautious regarding leucine, isoleucine, and valine, as they do not exhibit typical behaviour as 'essential' for nitrogen. Previous observations have noted fractionation in these amino acids (indicating their role as trophic amino acids). In all three cases, the diet-to-consumer offset was observed to be quite high, approximately 5 permille, with a significant level of uncertainty. For more insights, refer to experimental studies such as McMahon and McCarthy (2016).

  McMahon, K.W. and McCarthy, M.D., 2016. Embracing variability in amino acid δ15N fractionation: mechanisms, implications, and applications for trophic ecology. Ecosphere, 7(12), p.e01511.

- Page 11 (lines 315-317), regarding: “In order to assess quality control (QC), we used the relationship between proline and hydroxyproline since the hydroxylation of proline to form hydroxyproline does not involve exchange of nitrogen atoms [56]”.

  You should be careful here, as you only consider the quality of proline and hydroxyproline. Ideally, you should estimate the d13C and d15N bulk stable isotope values from the amino acid values and compare them with your measured bulk SIA values. See e.g. Fontanals-Coll, et al. (2023), but also older research by Styring and Howland. I consider this really important to assess the quality of your research, please try to address.

  Fontanals-Coll M, Soncin S, Talbot HM, von Tersch M, Gibaja JF, Colonese AC, Craig OE. Stable isotope analyses of amino acids reveal the importance of aquatic resources to Mediterranean coastal hunter-gatherers. Proc Biol Sci. 2023 Feb 22;290(1993):20221330. doi: 10.1098/rspb.2022.1330.

- Page 13 (lines 379 - 380), regarding “Previous studies have shown that the d13CGly-Phe values are an effective proxy for discriminating between terrestrial and marine sources [6, 7, 11, 58]”.

  The figure does not provide an explanation of how the shaded regions, within which terrestrial and aquatic values are expected to fall, were created. I assume you utilised data from these studies? If so, please make this explicit, perhaps in the figure's caption, to enhance clarity.

- Page 13 (lines 385-386), about: “Our d13CVal-Phe results show that d13CVal-Phe can separate terrestrial from aquatic sources.”

  Figure? or Figure 4B could be: axis x: Gly-Phe, axis y: Val-Phe rather than bulk d15N as it has been previously done.

- Page 14 (lines 394-396), regarding: “To assess differences in food groups, we carried out principal component analyses (PCA) with five d13C (Phe, Leu, Val, Ile, Thr) and four d15N (Val, Leu, Ile, Phe) values of essential amino acids.”

  Consistency is crucial here. Although you decided to run the PCA with essential amino acids, you included trophic amino acids. You should exclude Val, Leu, and Ile for their stable nitrogen isotope values for this. I suggest to re-run the PCA and decide between two different options: a) Repeat the

analysis but excluding d15N for Ile, Leu, and Val, or b) Consider the inclusion of all (essential, non-essential, trophic) amino acids. Both approaches will provide different insights; however, the first option (a), while not altering the results significantly, ensures consistency for readers and researchers in the field.

- Page 15 (lines 419-420), regarding: “A MixSIAR model was conducted with five d13CAA values (Leu, Val, Ile, Thr, Phe) and one d15NAA value (Phe).”

  I would like to once again encourage the authors to publish the scripts. It's not entirely clear how you constructed your MixSIAR model. Did you consistently take into account the calorific or protein contribution of different foodstuffs? For instance, did you consider the protein contribution of cereals, assuming 70% carbohydrates and 30% proteins? If so, I am satisfied with the model. However, I believe that, like me, other researchers would appreciate having this information available. This isn't just for the purpose of assessing your work but also for the benefit of learning and improving their own methodologies and interpretations , especially considering that CSIA-AA is still cutting-edge.
